# Liquid Type Nontoxic Photoluminescent Nanomaterials for High Color Quality White-Light-Emitting Diode

**DOI:** 10.1186/s11671-018-2835-4

**Published:** 2018-12-22

**Authors:** Chih-Hao Lin, Yung-Min Pai, Chun-Fu Lee, Akta Verma, Huang-Yu Lin, Chang-Ching Tu, Xin-Yin Chen, Hsi-Sheng Teng, Teng-Ming Chen, Cheng-Huan Chen, Chin-Wei Sher, Po-Tsung Lee, Chien-Chung Lin, S. K. Sharma, Hao-Chung Kuo

**Affiliations:** 1 Department of Photonics, National Chiao Tung University, 1001 University Road, Hsinchu, 300 Taiwan; 2 Department of Chemical Engineering, National Cheng Kung University, Tainan, Taiwan; 3 Department of Applied Chemistry, National Chiao Tung University, Hsinchu, Taiwan; 4 Institute of Photonic System, National Chiao Tung University, Tainan, Taiwan; 5 Hong Kong University of Science and Technology, Hong Kong, Hong Kong; 6 Department of Applied Physics, Indian Institute of Technology (Indian School of Mines), Dhanbad, 826004 India

**Keywords:** Quantum dots, Light-emitting diode, Non-toxicity, Graphene, Silicon

## Abstract

High-brightness white-light-emitting diodes (w-LEDs) with excellent color quality is demonstrated by using nontoxic nanomaterials. Previously, we have reported the high color quality w-LEDs with heavy-metal phosphor and quantum dots (QDs), which may cause environmental hazards. In the present work, liquid-type white LEDs composed of nontoxic materials, named as graphene and porous silicon quantum dots are fabricated with a high color rendering index (CRI) value gain up to 95. The liquid-typed device structure possesses minimized surface temperature and 25% higher value of luminous efficiency as compare to dispensing-typed structure. Further, the as-prepared device is environment friendly and attributed to low toxicity. The low toxicity and high R9 (87) component values were conjectured to produce new or improve current methods toward bioimaging application.

## Background

Light-emitting diodes (LEDs) have gained considerable attention because of their long-lifetime, high-efficiency, and energy-saving properties that make it a best candidate for solid-state lighting. GaN-based chips white LEDs (WLEDs) have demonstrated great improvements in efficiency due to fabrication progress [1–3]. GaN nitride base quantum dot (QD) technology has become highly suitable for various applications such as displays, light-emitting diode (LED) lighting, and biomedical labeling. In particular, numerous studies have been demonstrated excellent usage of QDs in white LEDs fabrication [4–6]. The characteristic features of QDs, such as their narrow emission linewidth, high quantum yield, and size-dependent tunable bandgap, made them excellent candidates toward LEDs technology [7–11]. The most common II–VI semiconductor QDs, such as cadmium and selenium compound cores (e.g., CdZnS, CdSe, CdZnSe, and ZnSe) with single or multiple shells based LEDs, have high luminous efficiency [12]. However, the high synthesis cost and heavy-metal toxicity of these QD materials hinder their large-scale production and raise concerns regarding environmental pollution [13]. The alternative materials based on silicon (Si) and carbon, such as graphene, are preferable nontoxic and environmentally friendly to human beings. Further, Si incorporated QDs exhibits significant light emission with high photoluminescence (PL) efficiencies under strong confinement [14–18].

Graphene oxide quantum dots (GQDs) possess the sp2 domain as a transport mobility intermediary as well as disordered sp3 hybridized carbon and oxygen atoms. Therefore, the emission wavelengths can be modulated from blue to green because of the presence of these disordered oxygen-containing edge states [19–22]. The tunable fluorescent emissions of QDs can be exploited for applications in devices such as LEDs, photodiodes, photodetectors, bioimagers, and photovoltaic cells [23–25]. In addition to the oxygen functionalization of GQDs, nitrogen doping can yield stable emission through the formation of covalent bonds with sp2 carbon in the aromatic chain. Nitrogen-doped GQDs that exhibit both p and n-type conductivities, as confirmed through electrochemical Mott–Schottky analysis, have been developed [26]. The main approaches to synthesize GQDs can be classified as top-down or bottom-up techniques. In comparison to the bottom-up approach, the top-down approach for GQDs production is more preferable for mass production as it does not require tedious purification steps for the removal of unreacted precursor molecules. However, the top-down approach produces a lower quantum yield (less than 50%) of GQDs than the bottom-up approach does [27, 28]. As a result, various optimizations are required in top-down fabrication processes of GQDs such as size control, chemical doping, or surface modification. The present study demonstrates a nitrogen-doped GQDs method to repair the defects that occur during a top-down process. Some electron-donating nitrogen functionalities can be incorporated into the GQDs and hydrothermal treatments with NH_3_ to avoid the formation of carbon-containing groups that might complicate the analysis of the nitrogen functionalities [29].

In this study, photoluminescence (PL) studies of nontoxic QD-based LEDs have been demonstrated by using GQDs and porous Si (P-Si) QDs. The PL-based QD LEDs offers a low-cost and simple fabrication approach over electroluminescent (EL) QD LEDs [30, 31]. Nitrogen-doped GQDs were used to manufacture nontoxic, neutral white LEDs. However, most of the GQDs emitted short-wavelength light (blue and green) under ultraviolet (UV) excitation. This is due to the quantum confinement effect (< 10 nm) that is normal to the graphene plane and shifting of emissions toward longer wavelengths by tuning the sizes of graphene crystals was difficult [32]. Therefore, Si QDs were embedded on the surfaces of P-Si nanoparticles, the defects of which resulted in fluorescence [33]. The P-Si nanocrystals can exhibit long-wavelength emissions to compensate for the absence of long-wavelength bands in the GQD emission spectra, and thus can yield warm white light. As per literature survey, L. T. Canham’s group contributed substantially to investigations of mesoporous Si layers with high porosity for visible (red) photoluminescence at room temperature [34]. The fabrication of P-Si QDs can be categorized into two approaches, bottom-up and top-down, similar to GQD fabrication processes. This study selects a top-down approach to fabricate the P-Si QDs because it is suitable for mass production. Finally, these two types of device structures neutral white and warm white LEDs were fabricated by using dispensing and liquid-type package processes were exhibited excellent color rendering index (CRI) and luminous efficiency values and also produces [34–36].

## Methods and Materials

### Synthesis of Nitrogen-Doped GQDs

First, graphene oxide (GO) sheets were prepared by using natural graphite powder (SP-1, Bay Carbon, USA) through the Hummers method which can be explained as follows [36]: 5 g of graphite powder, NaNO_3_, and KMnO_4_ were mixed in a 2:1:3 ratio in 150 mL of 18 M H_2_SO_4_ and the temperature of the mixture was maintained below 20 °C. The graphite was oxidized through continuous stirring of the mixture at 35 °C for 4 h, following which 230 mL of water was slowly added with stirring at 98 °C for 15 min. Subsequently, 12 mL of H_2_O_2_ was added to the mixture with continuous stirring at room temperature, and the product was washed several times in order to obtain GO sheets. The obtained GO sheets were doped with nitrogen by oxidizing them in 30 mL of NH_3_ (60% concentration) at 500 °C for 12 h. Then, the resulting mixture was ultrasonically agitated for 10 h, and was kept at 140 °C to remove residual HNO_3_. The final product was dispersed in deionized water and centrifuged to remove the precipitate. As a result, we obtained the nitrogen-graphene oxide dots (NGOD) suspensions [37]. These suspensions were sieved using centrifugation tubes (VS20S01 and VS15RH91, Satorius, Germany) to obtain GQDs. The centrifugation tubes were equipped with polyethersulfone filters with cut-off molecular weights of 30, 10, and 3 kDa to produce GQD3, GQD2, and GQD1, respectively. The GQD suspension was passed through membranes arranged in a sequence of decreasing pore size and collected at serial stages to obtain GQDs of varying sizes.

### Fabrication of P-Si QD Nanoparticles

Colloidal P-Si QD nanoparticles dispersed in ethyl acetate were synthesized as described in our recently published study [36–38]. First, a 6-in p-type Si wafer was electrochemically etched to produce a P-Si layer, on which clusters of nanosized (< 5 nm) P-Si QDs were attached to micro sized (1–10 μm) Si cores. The Si wafer was treated with diluted hydrogen fluoride (HF) and immediately immersed in deoxygenated 10-Undecen-1-ol (UDA) to complete a photochemical hydrosilylation reaction in which the terminal unsaturated double bond of UDA reacted with Si hydride (Si-H), resulting in Si-C bonded carboxylate passivation on P-Si QDs. Subsequently, the P-Si layer was mechanically pulverized, and the resulting Si powder was dispersed in isopropanol for high-energy ball milling. The colloid recovered from the milling was selectively etched using an aqueous etchant composed of HNO_3_ and HF for etching away the nonradiative bulk Si cores capped with the Si oxide while mostly preserving the PL P-Si QDs with the Si-C bonded carboxylate passivation. This step yielded approximately 25 mg per wafer of red-emitting hydroxyl-terminated P-Si QD nanoparticles (the actual Si QD is around 10 nm, approximately 40 nm to 500 nm in diameter) with high monodispersity and high PL quantum efficiency (45–55%). Finally, the hydroxyl groups of the P-Si QD nanoparticles were activated using p-toluenesulfonyl chloride and then substitution-reacted with 2,2′-(ethylenedioxy)diethanethiol to produce thiol-terminated P-Si QD nanoparticles. The resulting P-Si QD nanoparticles formed a uniform and stable suspension in ethyl acetate, which was used for optical characterization [38].

### Device Fabrication

**Fig. 1:**
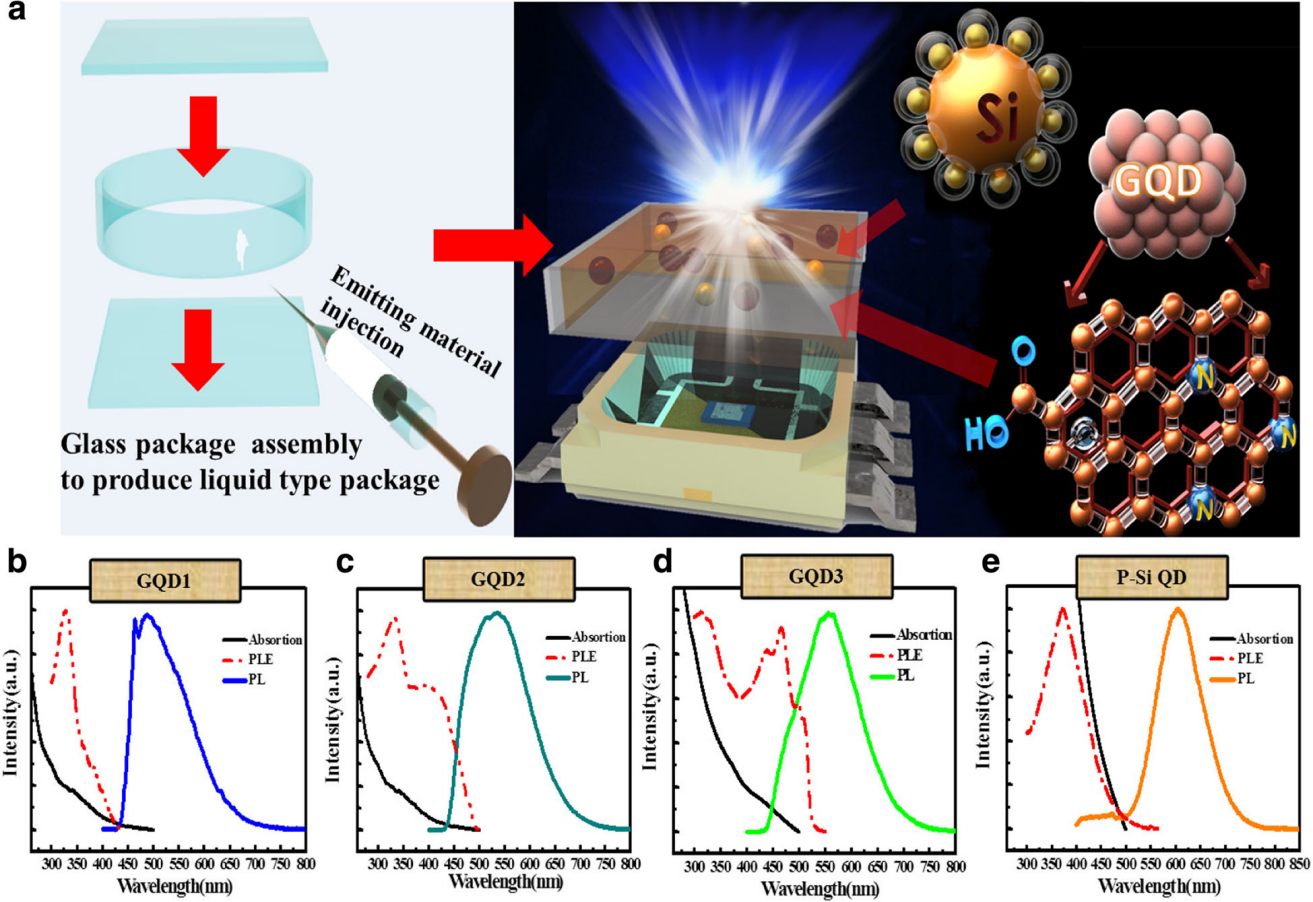
Top panel: **a** (left) glass package assembly showing a liquid-type P-Si QD and a nitrogen-doped GQD and (right) a network of P-Si QDs and nitrogen-doped GQDs. Bottom panel: absorption (black solid line), PL excitation (dashed line), and PL emission (solid line) spectra of **b** GQD1, **c** GQD2, **d** GQD3, and **e** P-Si QD

Two types of host structures, i.e., the dispensing structure and the liquid-type structure, were used to fabricate GQD and GQD/P-Si QD white LEDs. The fabricated structures were pumped by 45-mm UV (365 nm). At first, nitrogen-doped GQDs (wt% in water) with different emissions—blue, teal, and chartreuse—were prepared and denoted as GQD1, GQD2, and GQD3, respectively. Then, GQD1, GQD2, and GQD3 were mixed in different ratios (e.g., 4:1:2) to determine the optimal composition for obtaining neutral white emissions; the LED prepared using the GQD solution and the dispensing 5070 package was used as a reference. P-Si QDs were prepared and mixed with GQD1, GQD2, and GQD3 (GQD1:GQD2:GQD3: P-Si QD = 4:1:2:10) to fabricate white LEDs as sources of warm white light. Our previous study confirmed that the liquid-type structure is more favorable than the conventional structure [35]. In this study, we designed a new liquid-type structure to fabricate GQD and GQD/P-Si QD white LEDs. The GQD liquid-type neutral white LEDs were fabricated as follows: At first, we use a 2.5-mm-high glass ring with outer and inner diameters of 8 mm and 6 mm, respectively. After that, we drilled a small hole on the top surface of the glass ring. Finally, a glass box was assembled by stacking two thin glass plates with the glass ring in the middle (Fig. 1a). The space inside the glass box was left empty to promote air flow. Finally, GQD/P-Si QD solution was injected into the glass box to produce the liquid-type package. The QDs were injecting through the glass hole and sealed it with glass plate again. The liquid-type QD package was set on top of a 5070 UV LED package (5 mm × 7 mm), and the emission wavelength was 365 nm. The LIV curve spectrum indicated that the turn-on voltage was about 3 V which was shown in Fig. 4c. For the fabrication of the dispensing package, the conventional dispensing QD LEDs process was applied [34, 39]. In case of dispensing QD package approach, firstly we mixed the PMMA and QDs to produce the solidified structure in the LED package. For this, we filled the silicone glue half in the package in order to keep away the QD mixture from the heat source (blue chip) and prevent the QD degradation. After that, the volume ratio of our dispensing sample was taken as 2:1 of QD mixture/PMMA, and then dispensed the slurry to fill the remaining space in the package. After that the final structure was kept at 60 °C for 2–3 min for solidify, in this way, the PMMA/QD mixture film has been deposited in the LED package.

## Results and Discussion

**Fig. 2:**
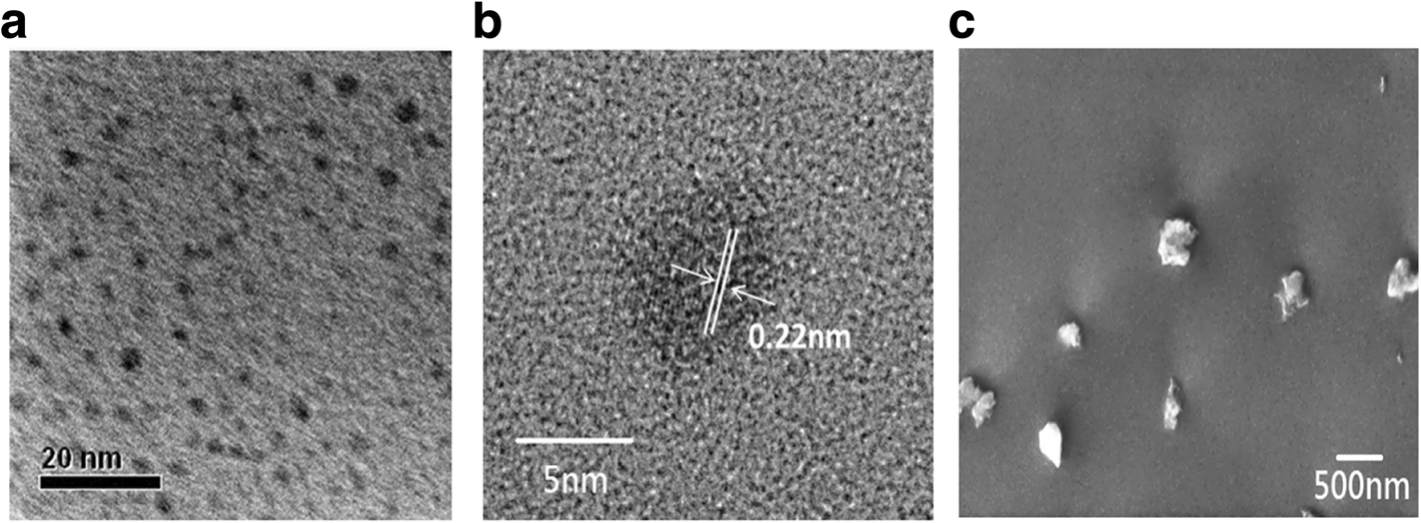
Transmission electron microscopy and scanning electron microscopy images of NPs. **a** Morphology of the graphene QD mixture with a particle size of approximately 5 nm as characterized through **a** TEM and **b** high-resolution TEM. **c** SEM image (top view) of the 40 nm–500 nm silicon particles

Figure 1a illustrates the network of P-Si QDs (left panel) and nitrogen-doped GQDs (right panel) and the liquid-type QD LED package. P-Si QDs with bio-probes can be fabricated using novel top-down methods, examples of which include electrochemical etching on a crystalline Si wafer [38, 40]. Figure 1b–e represents the absorption, PL excitation, and emission spectra of GQD1, GQD2, GQD3, and P-Si QDs. The black and red dashed lines indicate the absorption and excitation spectra of the QDs, respectively. The PL spectra of the investigated QDs covered a wide portion of the visible region. The full width at half maximum (FWHM) values of GQD1, GQD2, GQD3, and P-Si QDs were approximately 370, 325, 330, and 250 nm wavelengths, respectively. The strong emission bands were observed at 465 and 488 nm for GQD1 and at 535 nm for GQD2 after an excitation of 327 nm. An emission wavelength peak was observed at 557 nm attributed to GQD3 for two strong excitation peaks (311 and 465 nm), and a strong emission peak at 606 nm of P-Si QDs was aroused due to excitation peak at 374 nm. It can be depicted from the PL results that a shortwave pump was the preferred excitation source because the absorption and excitation of all luminaries were strongest in the UV region. Thus, a 365-nm UV LED was chosen as a suitable source to realize high conversion efficiency in the investigated QDs. The quantum yields of GQD1, GQD2, GQD3, and P-Si QDs at 365-nm excitation were approximately 1.4%, 1%, 9.1%, and 50%, respectively. The result shows that most of the GQD NPs were monolayer or bilayer, and the P-Si QDs were 40–500 nm in size approximately, indicating a multilayer composite structure. Figure 2a, b represents the transmission electron microscopy (TEM) and high-resolution TEM images that clarify the morphology and crystal structure of the GQD mixture. The size of the graphene QDs was found to be 5 nm, which corresponds to the spacing of ($1\overline{1}00$) plane and a lattice spacing of 0.22 nm [41]. Figure 2c is a scanning electron microscopy (SEM) image that shows the top-view of Si particles. The particles size was approximately in the range of 40–500 nm in size. Further, the few P-Si QDs were found approximately to 10 nm in size on the surfaces of Si particles.

**Fig. 3:**
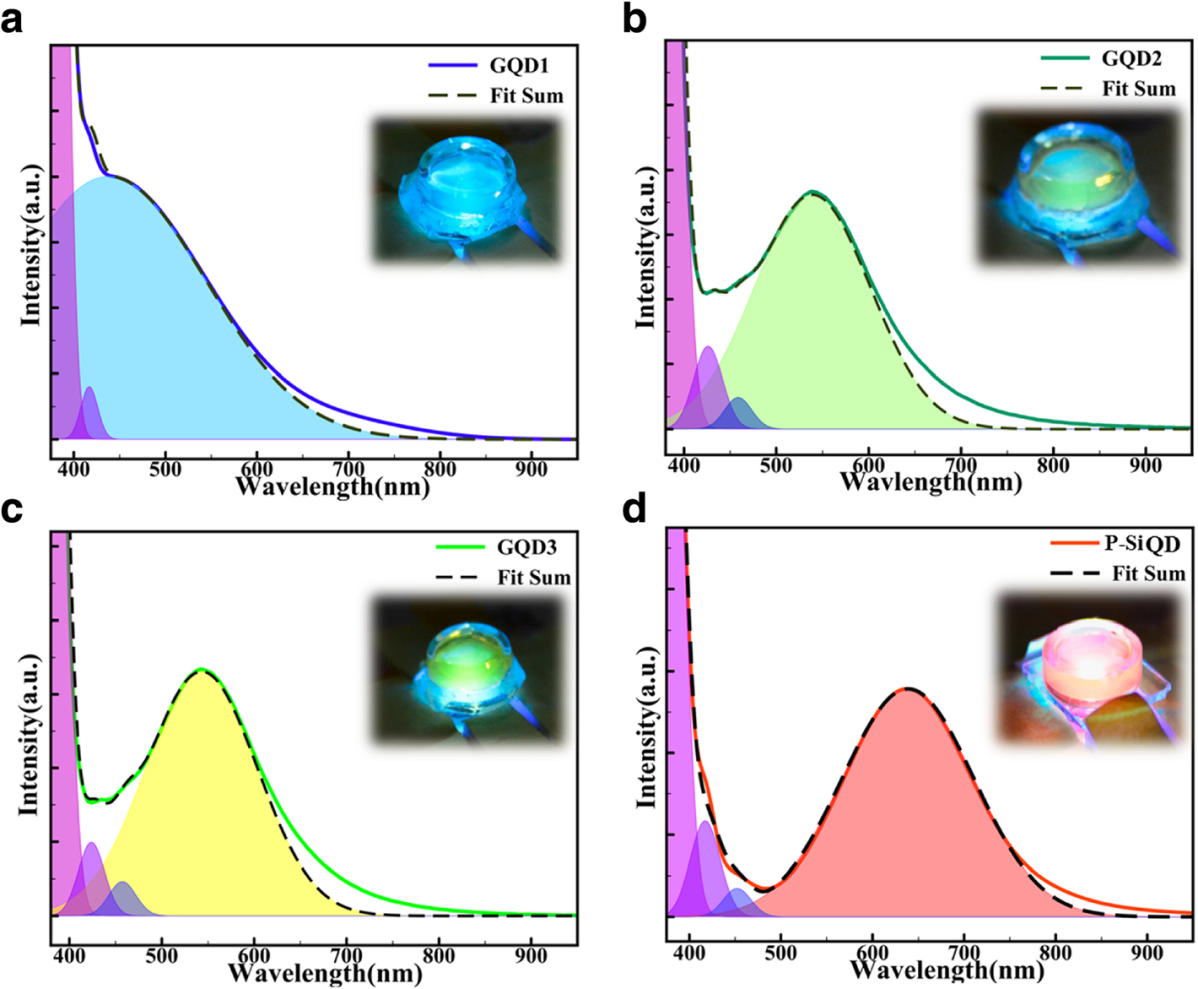
Spectra of the liquid-type monochrome QD LED package for **a** GQD1, **b** GQD2, **c** GQD3, and **d** P-Si QD at a current of 60 mA

The LEDs that emitted high-quality white light in this study composed of several nanomaterials with different emission peak as to cover wide range colors. To investigate the monochromaticity of these emitting materials, the liquid-type GQD1, GQD2, GQD3, and P-Si QD LED packages were pumped using a 365-nm UV chip, and their emission spectra were recorded in Fig. 3. The spectra of GQD1 exhibited an emission peak at 440 nm and covered a large portion of the blue wavelength region to yield blue rays was shown in Fig. 3a. The blue wavelength region in the spectrum of GQD2 was slightly smaller than that of GQD1. Consequently, liquid-type GQD2 emitted teal light with an emission peak at 538 nm (Fig. 3b). The emission spectra of liquid-type GQD3 had a strong yellow peak (550 nm), which caused GQD3 to emit chartreuse light shown in Fig. 3c. After UV pumping, Fig. 3d shows that the liquid-type P-Si QD package emitted orange rays with a strong peak located at 636 nm. The monochromaticity of QD LEDs demonstrated different wavelengths with noticeable changes for the PL analysis (compare to Fig. 1b–d). The main cause of the different emission wavelengths was the different pumping source. A 365-nm UV LED was used as excitation lighting source; this involved an excitation wavelength of 327 nm for GQD1 and GQD2, a wavelength of 311 nm for GQD3, and a wavelength of 374 nm for P-Si QDs [42, 43]. After forming composite mixtures, all graphene and P-Si QDs exhibited broad spectral bands that generated high-quality white light with high CRI values.

**Fig. 4:**
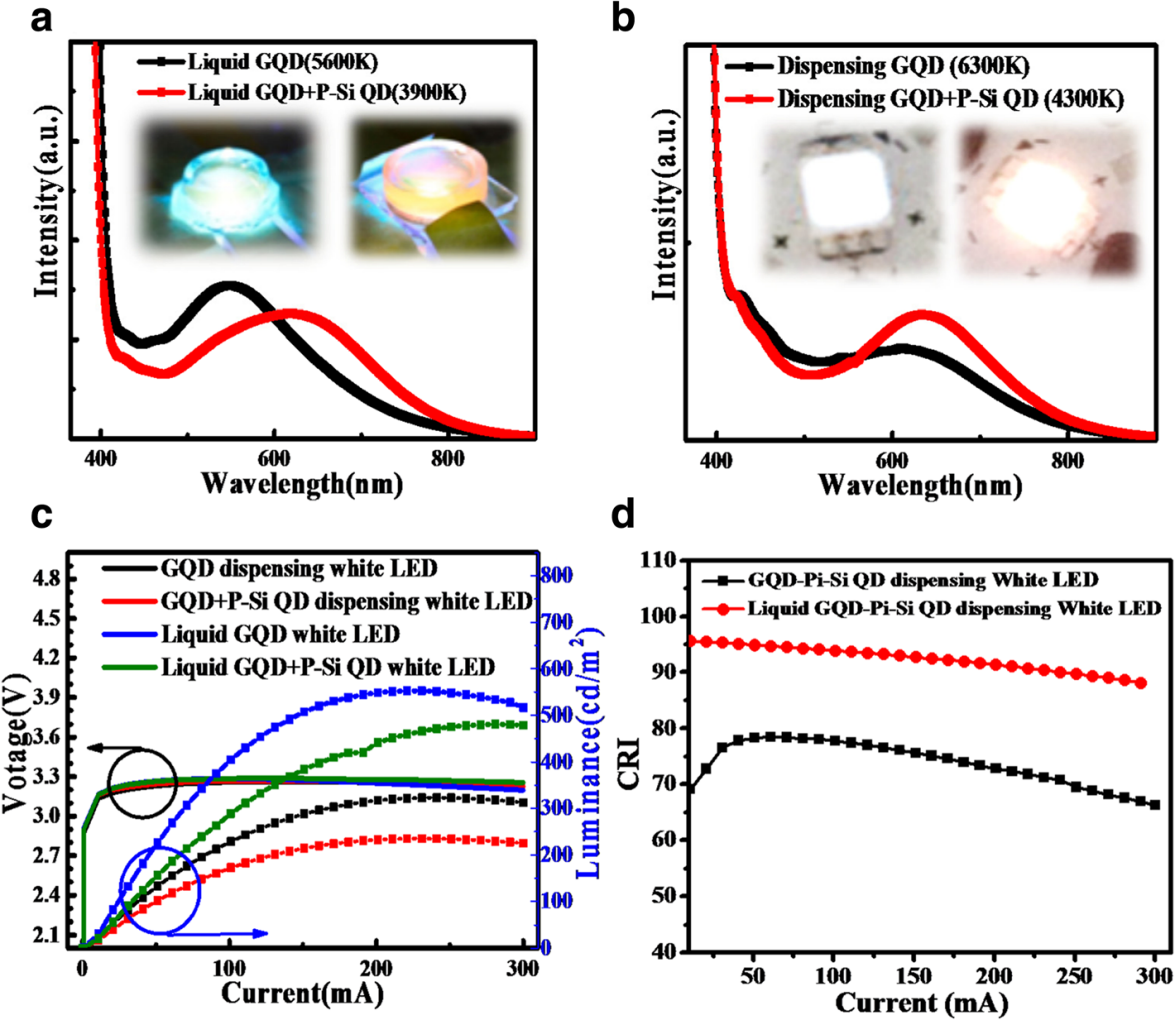
PL spectra of (**a**) liquid-type graphene QD at a CCT of 5600 K and liquid-type GQD/ P-Si QD white LED at a CCT of 3900 K. **b** Dispensing graphene QD and graphene/P-Si QD white LEDs. **c** The LIV curve spectrum of the nontoxic w-LEDs driven at 1–300 mA. **d** CRI spectra for GQD + P-Si dispensing and liquid-type LEDs driven at 1–300 mA

Figure 4a, b shows the spectra of dispensing and liquid-type white PL QD LEDs at 60 mA. The GQD liquid-type LED provided neutral white light at a correlated color temperature (CCT) of 5600 K with a luminous efficiency of approximately 20.3 lm/W; the emission spectra consist a peak centered at 548 nm approximately. The GQD/P-Si QD liquid-type LED device provided a warm white light with a CCT of 3900 K and a luminous efficiency of approximately 19.1 lm/W with an emission peak located at 625 nm in Fig. 4a. The dispensing samples fabricated using the GQD solution and the GQD/P-Si QD mixtures exhibited CCT values of 6300 and 4300 K and emission peaks were obtained at approximately 642 nm and 611 nm wavelengths, respectively. The obtained luminous efficiency values were found to be 16.2 lm/W and 14.5 lm/W for GQD neutral white LEDs and GQD/P-Si QD for warm white LEDs, respectively. In comparison to the liquid-type sample, the PL emission peaks of the dispensing samples are red-shifted because of QD self-aggregation, which is caused due to the absence of a carrier solution [44–46]. Further, the small particles are aggregate into larger particles, diversifying energy transfer [47–50]. On the other hand, the use of a mixture of QDs may cause the unintended energy transfer between different color QDs. The excellent CRI of a white LED could be modulated by energy transfer phenomenon, but caused the luminous reduction [51]. If we expected to prevent the unintended energy transfer, the side by side structure liquid-type QD LED fabricated by printing could be planned in the future, which was refer to the research from M. K. Choi et al [52]. The LIV curve spectra of nontoxic w-LEDs are plotted in Fig. 4c. The maximum output luminance of the w-LEDs was about 552 cd/m^2^ at 230 mA for the liquid-type CQD w-LED, and the dispensing samples had lower luminance values. The turn-on voltage was about 3 V, and all samples were driven with similar input power. Figure 4d presents the CRI of GQD/P-Si QD dispensing and liquid-type samples at current injection values ranging from 1 to 300 mA. The liquid-type package was more stable and prevented the self-aggregation and the spectral red shift, which maintained the CRI. We modified the ratio of QD mixing to achieve this excellent color quality. The warm white liquid-type LEDs had an excellent CRI of 95. The liquid-type samples exhibit a higher general CRI (Ra) value compared to the dispensing samples. Further, the lower CRI of the dispensing samples is attributable to the QD self-aggregation and the spectral red shift of the dispensing sample. The absence of yellow and green emissions and the enhancement of orange and red emissions decreased Ra [32]. When Ra was not decreased, the use of the liquid-type package was able to maintain the shapes of the emission spectra. The CIE chromaticity coordinates of the liquid-type and dispensing samples were close to the Planckian locus. The CRI values for R1–R9 follow the decreasing trend. This is due to the red-shift phenomenon that occurred after the dispensing process. The liquid-type GQD/P-Si QD white LED exhibited excellent R9 (88) at 3900 K. The high R9 values are desirable because of their association with strong red emissions, which are related to organic tissues [53]. On the basis of CRI values, it can be depicted from here that the liquid-type samples are better than the dispensing samples. The dispensing sample has low value of CRI because of self-aggregation, intensity reduction, and red shift of the conformal QD LEDs. Further, various studies of graphene QD LEDs have been published. But still there are only few studies that demonstrated the CRI values of QD LEDs. Hence, in the present study, QD-based WLEDs were fabricated with excellent CRI values, as shown in Table 1.

**Table 1:** The values of CRI No. 1 to No. 15 (R1–R15) and CRI of the dispensing and liquid-type warm white and neutral white QD LEDs

	R1	R2	R3	R4	R5	R6	R7	R8	R9	R10	R11	R12	R13	R14	R15	Ra
Dispensing GQD/PQD	78	83	95	75	75	76	96	73	37	72	75	55	75	96	63	81
Liquid GQD/PSQD	97	95	94	92	96	93	98	95	87	90	96	89	92	96	92	95

**Fig. 5:**
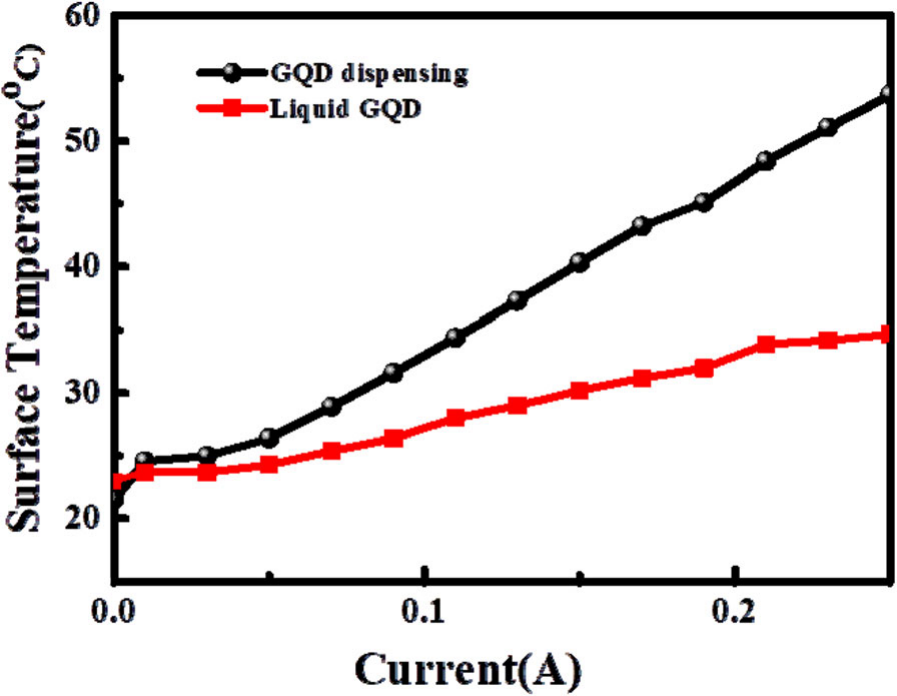
Average surface temperature and current dependence of liquid-type and dispensing white LEDs

Figure 5 represents the average surface temperature and current dependence of liquid-type and dispensing white LEDs. The current-dependent surface temperatures were measured as the average temperature over the device surface area, for a period of 2 min, with the device driven from 1 to 250 mA. Out of the two prepared structures, the dispensing samples exhibited the lower luminous efficiencies and the higher surface temperatures; this is attributed to heat trapping within the package. Equation (1) was used to calculate the heat dissipation in the device as the difference between the input electrical power and the measured light intensity:

1 $$ {P}_{heat}={P}_{elec.}-{P}_{opt.}={I}_f{XV}_f-\frac{{\mathrm{\O}}_v}{Wpot.} $$

where P_elec._ is the injected electrical power; P_heat_ and P_opt._ are the generated heat energy and optical power after the input power is injected, respectively; I_f_ and V_f_ are the drive current and forward voltage at LED operating conditions, respectively; Ø_v_ is the total luminous flux; and W_opt_ is the luminous efficacy of optical radiation (LER) of the LEDs. The primary reason for the difference in the surface temperature of these packages is the difference in their thermal conductivity coefficients: 1.05 W/mK for liquid-type samples, which are composed of glass, and 0.185–0.196 W/mK for the dispensing samples, which are composed of PMMA. The glass container of the liquid-type samples facilitates heat dissipation and thus has a high luminous efficiency. Thus, improving the heat dissipation characteristics of the samples can enhance the photon output.

## Conclusions

In summary, we have prepared two types of WLEDs device structures one is dispensing structure and the other is liquid-type structure by using GQD and GQD/P-Si QD respectively. The graphene QDs and porous silicon QDs have extremely wide emission bands. The obtained results indicate that graphene QDs and silicon nanocrystals-based w-LEDs possess excellent values of CRI (95) and R9 (88). Further, the liquid-type device structure exhibits higher luminous efficiency by 25% and better stability as compared to dispensing structured devices. Finally, we can conclude that the excellent performance of the nontoxicity liquid-type warm LEDs have the great potential in bioimaging and other related application such as lighting and sensing are of great interest.

## Abbreviations

CRI: Color rendering index
GO: Graphene oxide
GQDs: Graphene oxide quantum dots
LED: Light-emitting diode
PL: Photoluminescence
QDs: Quantum dots
W-LEDs: White-light-emitting diodes
